# Estimating Global “Blue Carbon” Emissions from Conversion and Degradation of Vegetated Coastal Ecosystems

**DOI:** 10.1371/journal.pone.0043542

**Published:** 2012-09-04

**Authors:** Linwood Pendleton, Daniel C. Donato, Brian C. Murray, Stephen Crooks, W. Aaron Jenkins, Samantha Sifleet, Christopher Craft, James W. Fourqurean, J. Boone Kauffman, Núria Marbà, Patrick Megonigal, Emily Pidgeon, Dorothee Herr, David Gordon, Alexis Baldera

**Affiliations:** 1 Nicholas Institute for Environmental Policy Solutions, Duke University, Durham, North Carolina, United States of America; 2 Ecosystem & Landscape Ecology Lab, University of Wisconsin, Madison, Wisconsin, United States of America; 3 ESA Phillip Williams & Associates, San Francisco, California, United States of America; 4 United States Environmental Protection Agency, Research Triangle Park, North Carolina, United States of America; 5 School of Public and Environmental Affairs, Indiana University, Bloomington, Indiana, United States of America; 6 Department of Biological Sciences and Southeast Environmental Research Center, Florida International University, North Miami, Florida, United States of America; 7 Department of Fisheries and Wildlife, Oregon State University, Corvallis, Oregon, United States of America and Center for International Forest Research, Bogor, Indonesia; 8 Department of Global Change Research, Mediterranean Institute for Advanced Studies, Esporles, Illes Balears, Spain; 9 Smithsonian Environmental Research Center, Edgewater, Maryland, United States of America; 10 Conservation International, Arlington, Virginia, United States of America; 11 International Union for the Conservation of Nature, Washington, District of Columbia, United States of America; 12 The Ocean Conservancy, Baton Rouge, Louisiana, United States of America; National Institute of Water & Atmospheric Research, New Zealand

## Abstract

Recent attention has focused on the high rates of annual carbon sequestration in vegetated coastal ecosystems—marshes, mangroves, and seagrasses—that may be lost with habitat destruction (‘conversion’). Relatively unappreciated, however, is that conversion of these coastal ecosystems also impacts very large pools of previously-sequestered carbon. Residing mostly in sediments, this ‘blue carbon’ can be released to the atmosphere when these ecosystems are converted or degraded. Here we provide the first global estimates of this impact and evaluate its economic implications. Combining the best available data on global area, land-use conversion rates, and near-surface carbon stocks in each of the three ecosystems, using an uncertainty-propagation approach, we estimate that 0.15–1.02 Pg (billion tons) of carbon dioxide are being released annually, several times higher than previous estimates that account only for lost sequestration. These emissions are equivalent to 3–19% of those from deforestation globally, and result in economic damages of $US 6–42 billion annually. The largest sources of uncertainty in these estimates stems from limited certitude in global area and rates of land-use conversion, but research is also needed on the fates of ecosystem carbon upon conversion. Currently, carbon emissions from the conversion of vegetated coastal ecosystems are not included in emissions accounting or carbon market protocols, but this analysis suggests they may be disproportionally important to both. Although the relevant science supporting these initial estimates will need to be refined in coming years, it is clear that policies encouraging the sustainable management of coastal ecosystems could significantly reduce carbon emissions from the land-use sector, in addition to sustaining the well-recognized ecosystem services of coastal habitats.

## Introduction

Anthropogenic contributions to atmospheric greenhouse gases (GHG) are due largely to the combustion of fossil fuels. Land-use activities, especially deforestation, are also a major source of GHG, accounting for ∼8–20% of all global emissions [1]. While the role of terrestrial forests as a source and sink of greenhouse gases is well known, new evidence indicates that another source of GHG is the release, via land-use conversion, of carbon (C) stored in the biomass and deep sediments of vegetated ecosystems such as tidal marshes, mangroves, and seagrass beds. These coastal carbon stocks are increasingly referred to as “blue carbon” [2], [3]. The exact amount of carbon stored by these ecosystems is still an active area of research, but the potential contribution to GHG from their loss is becoming clear. Yet these emissions are so far relatively unappreciated or even neglected in most policies relating to climate change mitigation [4]. Here, we estimate the potential magnitude and economic impact of these previously unaccounted emissions.

**Figure 1:**
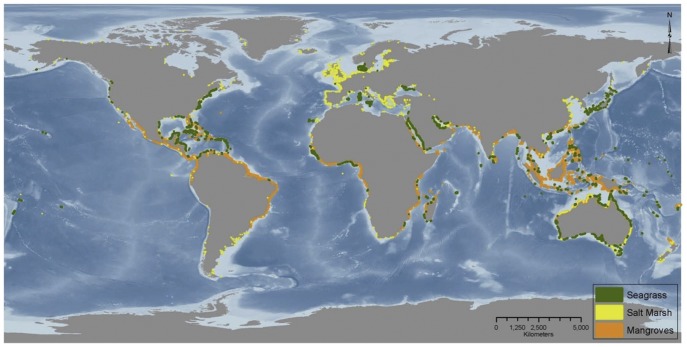
Global distribution of seagrasses, tidal marshes, and mangroves. Data sources: Seagrass and saltmarsh coverage data are from the United Nations Environment Programme World Conservation Monitoring Centre (UNEP-WCMC); mangrove coverage data are from UNEP-WCMC in collaboration with the International Society for Mangrove Ecosystems (ISME).

Carbon is stored in vegetated coastal ecosystems throughout the world (Figure 1). Seagrass beds are found from cold polar waters to the tropics. Mangroves are confined to tropical and sub-tropical areas, while tidal marshes are found in all regions, but most commonly in temperate areas. Combined, these ecosystems cover approximately 49 million hectares (Figure 1, Table 1) and provide a diverse array of ecosystem services such as fishery production, coastline protection, pollution buffering, and high rates of carbon sequestration [5].

**Table 1:** Estimates of carbon released by land-use change in coastal ecosystems globally and associated economic impact.

	Inputs			Results	
Ecosystem	Global extent (Mha)	Current conversion rate (% yr^−1^)	Near-surface carbon susceptible (top meter sediment+biomass, Mg CO_2_ ha^−1^)	Carbon emissions (Pg CO_2_ yr^−1^)	Economic cost (Billion US$ yr^−1^)
Tidal Marsh	2.2–40 (5.1)	1.0–2.0 (1.5)	237–949 (593)	**0.02–0.24 (0.06)**	**0.64–9.7 (2.6)**
Mangroves	13.8–15.2 (14.5)	0.7–3.0 (1.9)	373–1492 (933)	**0.09–0.45 (0.24)**	**3.6–18.5 (9.8)**
Seagrass	17.7–60 (30)	0.4–2.6 (1.5)	131–522 (326)	**0.05–0.33 (0.15)**	**1.9–13.7 (6.1)**
Total	33.7–115.2 (48.9)			**0.15–1.02 (0.45)**	**6.1–41.9 (18.5)**

Notes: 1 Pg = 1 billion metric tons. To obtain values per km^2^, multiply by 100. See Methods section for detailed description of inputs and their sources. In brief, data for global extent and conversion rate are recently published ranges (minimum - maximum, and central estimate in parentheses). For near-surface carbon susceptible to land-use conversion (expressed in potential CO_2_ emissions [48]–[50]), uncertainty range is based on assumption of 25–100% loss C upon land-use impact; thus, the high-end estimate is the literature-derived global mean carbon storage in vegetation and the top meter of sediment only (central estimate is thus 63% loss). Results for carbon loss are non-parametric 90% confidence intervals (median in parentheses) from Monte Carlo uncertainty propagation of the three input variables (see Methods). Economic estimates apply a multiplier of US$ 41 per ton of CO_2_ to lower, upper, and central emission estimates (see Methods).

Rapid loss of vegetated coastal ecosystems through land-use change has occurred for centuries, and has accelerated in recent decades. Causes of habitat conversion vary globally and include conversion to aquaculture, agriculture, forest over-exploitation, industrial use, upstream dams, dredging, eutrophication of overlying waters, urban development, and conversion to open water due to accelerated sea-level rise and subsidence [6]–[12]. Estimates of cumulative loss over the last 50–100 years range from 25–50% of total global area of each type [12]. This decline continues today, with estimated losses of ∼0.5–3% annually depending on ecosystem type, amounting to ∼8000 km^2^ lost each year [7], [11], [13]–[19]. At current conversion rates, 30–40% of tidal marshes and seagrasses [20] and nearly 100% of mangroves [8] could be lost in the next 100 years.

An emerging body of literature recognizes the importance of coastal habitat loss to climate change [2], [15], [21], [22]. However this research has focused almost exclusively on the lost carbon sequestration potential (annual uptake), while the conversion of large standing carbon pools (previously sequestered and stored C) associated with vegetated coastal ecosystems has been relatively overlooked. Only in the most recent studies and reviews has the release of standing carbon pools begun to gain more attention [12], [23], [24].

Quantitative estimates of these emissions are scarce. Indications are that such ‘pulse’ releases may have the largest and most immediate impact on green house gas (GHG) emissions, possibly amounting to 50 times the annual net carbon sequestration rate [12], [25]. Similar greenhouse gas emissions from the conversion or degradation of freshwater wetlands (e.g., peatlands) are recognized by scientists and international policy-making bodies [1], [26], while blue carbon remains largely unaccounted.

Vegetated coastal ecosystems typically reside over organic-rich sediments that may be several meters deep and effectively ‘lock up’ carbon due to low-oxygen conditions and other factors that inhibit decomposition at depth [27]. These C stocks can exceed those of terrestrial ecosystems, including forests, by several times [24], [28]. When coastal habitats are degraded or converted to other land uses, the sediment carbon is destabilized or exposed to oxygen, and subsequent increased microbial activity releases large amounts of GHG to the atmosphere or water column [25], [27], [29]–[32]. For example, sediment C was reduced by 50% within 8 years after land clearing in a Panamanian mangrove [29]. Lovelock et al. [33] reported large short-term CO_2_ efflux from the sediment surface of cleared mangroves of approximately 29 Mg CO_2_ ha^−1^ yr^−1^. Eventually the majority of carbon in disturbed coastal ecosystems can be released to the atmosphere (in the form of CO_2_, CH_4_, or other carbon species) with the timeframe highly variable and dependent on the specific land use and nature of the sediment [23].

The potential economic impacts that come from releasing stored coastal blue carbon to the atmosphere are felt worldwide. Economic impacts of GHG emissions in general stem from associated increases in droughts, sea level, and frequency of extreme weather events [34]. Costs are believed to be borne most acutely in low-income countries. However, the potentially large carbon emissions from degraded vegetated coastal ecosystems may also offer a new carbon mitigation opportunity that is currently unrealized—similar to, or even part of, Reduced Emissions from Deforestation and Degradation (REDD+), in which economic incentives encourage the maintenance of forest ecosystem C storage [4].

Our objective here is to provide the most comprehensive estimate to date of the global carbon emissions and economic impacts of the ongoing conversion of standing carbon stocks in coastal ecosystems, including carbon emissions from sediments – the first analysis to do so. Policy makers need at least an order of magnitude estimate of the potential importance of coastal habitat change as a contributor to global GHG emissions. Although uncertainties exist in the available data underlying such estimates, there is strong need at the international level for the most up-to-date assessment; sufficient information is available to evaluate the importance of coastal blue carbon in both absolute and relative terms. Given the scientific uncertainties present, we used a parsimonious uncertainty/sensitivity framework to a) establish bookends that very likely contain the true value of global emissions from coastal ecosystem conversion, and b) identify the key data gaps relevant to moving forward with their inclusion in carbon policies.

## Methods

### Analytical framework

To gauge potential carbon emissions from the conversion of coastal ecosystems, we combined estimates of global area, current conversion rate (% of area lost per year), and near-surface carbon stocks susceptible to loss in each of the three habitat types (Table 1). Each of the input multipliers has varying degrees of uncertainty owing to ranges reported in the literature or limited available data. Therefore we used a Monte Carlo approach [35] to propagate uncertainties in each factor using the best available ranges from the literature (see below). Simulations comprised 50,000 iterations for each habitat type and assumed a normal distribution of input variables within reported ranges, except when ranges were heavily right skewed (i.e., a minority of extremely high estimates in the literature for a given input). In the latter case, we applied a simple gamma distribution with parameters corresponding to the minimum and maximum reported values in order to account for, but avoid undue influence of, possible high-end extremes. This distribution applied to global area estimates of tidal marsh (gamma shape 1.6, scale 6) and seagrass (shape 4, scale 4). Fifth and 95^th^ percentiles were extracted from the 50,000 Monte Carlo iteration outputs to obtain non-parametric 90% confidence intervals for emissions in each type.

### Data inputs

#### Global area

Global area inputs were derived from international monitoring databases and recently published literature. For tidal marshes, we applied a central estimate of 5.1 Mha (obtained from the United Nations Environment Programme-World Conservation Monitoring Centre [UNEP-WCMC] spatial data, 2005) and a range of 2.2 to 40 Mha [12], [15], [21]. The low-end estimate may be too low, but until improved estimates are available, we took the conservative approach of including the full range of data sources. It should also be noted that, in some cases, these published estimates were derived from the same primary sources, so not all values are truly independent of each other. Of the three habitat types considered, mangroves have perhaps the best global extent data and a fairly narrow range of reported values; we applied the recently reported range of 13.8 to 15.2 Mha [12], [19], [36] and a corresponding central estimate of 14.5 Mha. For seagrasses, we applied a central estimate of 30 Mha (obtained from UNEP-WCMC spatial data, 2005) and a range of 17.7 to 60 Mha [12], [37]–[39].

#### Annual area loss

Current rates of global annual loss (land-use conversion) were derived from recently published literature. We assigned a global annual loss rate of 1–2% for tidal marshes [12], [16], [18]; 0.7–3% for mangroves [7], [12], [14], [17], [19]; and 0.4–2.6% for seagrasses [11]–[13], [15], [38].

#### Carbon loss upon conversion

Carbon loss per hectare converted has not been well quantified in coastal ecosystems, but likely bounds can be derived. The loss of vegetation biomass is the most common and readily apparent result of conversion, but there are also losses from the surface sediment carbon pool (<1 m deep; [29], [32]) as well as potentially large, but not well understood, C losses from deep sediments [23], [40]. We therefore took a conservative approach by focusing only on carbon in vegetation and the top meter of sediment. These pools are most susceptible to land-use change and are termed here ‘near-surface’ carbon. For the uncertainty range used in the simulations, we used the best available estimate of global mean near-surface C in each ecosystem type, with a range of possible fates of this pool upon conversion, from 25% to 100% emission to the atmosphere depending on disturbance type, possible re-burial of disturbed material, and degree of C recalcitrance. The high end of 100% would apply if most land uses tend toward extreme impacts that convert the system to a qualitatively different state that removes and prevents recovery of near-surface carbon. The low end of 25% would apply if most land uses are relatively light-handed and retain, bury, or merely redistribute most near-surface carbon. Blue carbon that is oxidized via disturbance and exposure (converted to species such as CO_2_, HCO_3_^−^, or CO_3_^2−^) increases the effective CO_2_ concentration of the ocean-atmosphere system. Because of the partial pressure equilibrium of CO_2_ between air and water, atmospheric CO_2_ levels are affected by either direct ocean-to-atmosphere gas exchange, or by reductions in the ability of the ocean to absorb atmospheric CO_2_ [41].

Mechanisms of disturbance to sediment carbon vary by ecosystem type, but often affect near-surface carbon to at least one meter depth. In tidal marshes, a primary land-use activity is the creation of arable land via diking and draining, an effect that may persist for decades and lead to the loss of several meters of sediment, along with its carbon, due to oxidation [23]. For mangroves, conversion to aquaculture is widespread, with the excavation of mangrove sediments to depths of about one meter exposing a large portion of the sediment carbon to oxygen; system degradation through over-harvest can also lead to sediment erosion and exposure [25]. In seagrass systems, water quality impairment, generally from excess nutrients or sediments from terrestrial sources, is a leading cause of ecosystem decline and loss, and ultimately exposure of sediment carbon to the water column or atmosphere [42]. Direct impacts such as dredging, trawling, and anchoring also affect seagrass beds [42].

For near-surface carbon stocks (including just the top meter of sediment), studies suggest conservative carbon storage estimates of approximately 250 Mg of carbon per hectare for tidal marshes [16], [21]; 280 Mg C ha^−1^ for mangroves [24], [28], [43]; and 140 Mg C ha^−1^ for seagrasses [44]–[47]. Following IPCC protocol for tracking changes in carbon stocks [48]–[50], and to facilitate comparison among most other assessments, we express ecosystem carbon in terms of potential CO_2_ emissions – obtained by multiplying C stocks by 3.67, the molecular weight ratio of CO_2_ to C. The values for tidal marshes, mangroves, and seagrasses therefore become 917, 1028, and 512 Mg of potential CO_2_ emissions per hectare, respectively. These estimates are conservative since larger amounts of carbon are often held in as much as 6 meters of sediment and biomass beneath the emergent vegetation [21], [24], [51]. The carbon in emergent living biomass of these ecosystems ranges widely, from estimated mean values of 1 to 129 Mg C ha^−1^ (2 to 474 Mg of potential CO_2_ emission ha^−1^) depending on habitat type [16], [24], [28], [43], [45], [52]–[54]. This vegetation biomass increases the near-surface carbon estimates to global means of 259, 407, and 142 Mg C ha^−1^ (949, 1492, and 522 Mg of potential CO_2_ emissions ha^−1^) for tidal marshes, mangroves, and seagrasses, respectively (Table 1).

For each ecosystem, we focus on the total amount of CO_2_ that could be released from annual rates of conversion, but we do not attempt to estimate over what course of time these releases would be made. (At the scale of the individual site, the rate of release likely follows a negative exponential curve with time—initially high and tapering in later years. The temporal dynamic of near-surface carbon pools after conversion is a significant research need, but some studies suggest it may have a half-life on the order of 5–10 years [29].) It is important to note that any assumption of the temporal period of release within a degraded site, whether 5–10 years or much longer, is inconsequential to the results of this analysis. When summed over the globe and integrated over time, as long as ecosystem conversion rates are stable or increasing over time, the total amount of carbon released annually would be greater than or equal to our estimates.

### Conservative approach

We emphasize that the analysis above should be considered conservative in its estimate of emissions. First, we reduced the emphasis on high-end estimates of global area by using gamma distributions to minimize the impact of especially high estimates. Second, we did not include any potential impacts on deep sediment C (>1 m depth), in part because of limited available science. These layers often contain more C per hectare than all the near-surface carbon combined [24] and have been found to be impacted by land-use change in the few cases studied [23]. This means that even our high-end scenario of 100% C loss upon conversion is actually much less than all of the ecosystem carbon. Third, the low-end scenario of 25% C loss upon conversion effectively assumes that all land-use changes in coastal systems across the entire globe could retain 75% of all near-surface carbon (if most C in disturbed systems is merely buried or redistributed) – an extremely conservative assumption. Fourth, we did not include the loss of annual sequestration of sediment carbon that occurs due to vegetation removal or hydrological isolation that reduces new sediment inputs.

Regarding other greenhouse gases such as methane (CH_4_) or nitrous oxide (N_2_O), excluding changes in these components is likely either a neutral or conservative approach. In highly saline wetlands (>18 ppt), sediment C sequestration rates exceed CH_4_ emission rates in CO_2_-equivalent units [55], suggesting that the net effect of losing both sequestration and CH_4_ emissions with disturbance should be an increase in greenhouse gas emissions. In lower salinity wetlands (salinity 5–18 ppt), CH_4_ emissions and sequestration are approximately in balance [56], except perhaps for oligohaline systems (<5 ppt) that are a small portion of the global area we evaluated. Finally, we conservatively did not consider evidence that common disturbances, such as conversion to shrimp ponds, that cause eutrophication have been shown to stimulate CH_4_ emissions [27]. Eutrophication is likely to also increase N_2_O emissions if the system receives high nitrate loading; otherwise it is not necessary to account for changes in N_2_O fluxes because emissions from anaerobic sediments are negligible in the absence of nitrate loading.

### Economic impact

Finally, we calculated the estimated cost to the global economy of the estimated emissions resulting from coastal ecosystem conversion. We multiplied the global emissions estimates for each type by a recent estimate of the global economic cost of new atmospheric carbon of $41 per ton of CO_2_ (2007 U.S. dollars) [57]. This cost is a central estimate of the “social cost of carbon” (SCC), which is defined as the marginal value of economic damages of the climate change attributable to an additional ton of CO_2_ in the atmosphere in 2020 (2007 dollars) [57]. The SCC estimate is an estimate of the environmental damages that can be avoided by reducing emissions, but does not necessarily equal the price that the market will pay for reducing emissions, since that market price is determined by the avoided cost of regulatory controls on carbon and not avoided damages per se [57].

## Results and Discussion

### CO_2_ emissions

We estimate that the conversion and degradation of coastal ecosystems each year may ultimately release between 0.15 and 1.02 Pg (billion tons) of CO_2_ to the atmosphere, with a central estimate of 0.45 Pg CO_2_ (Table 1). Mangroves contain the largest per-hectare carbon stocks and contribute approximately half the estimated total blue carbon emissions. Seagrasses, despite containing the lowest per-hectare carbon stocks, contribute the second most to global blue carbon emissions, due to their larger global area. Tidal marshes contain moderate to high carbon stocks, but their relatively small total area results in the lowest—although still substantial—global emissions.

To put these emissions in perspective, the central estimate of 0.45 Pg CO_2_ yr^−1^ approaches the annual fossil fuel CO_2_ emissions of the United Kingdom (the world's 9^th^ ranked country by emissions), while the low estimate of 0.15 Pg is roughly equivalent to those of Venezuela (ranked 30^th^) and the high estimate of 1.02 Pg approaches those of Japan (ranked 5^th^) [20]. Comparing to other ecosystem C fluxes, the loss of vegetated coastal ecosystems may contribute an additional 3–19% above the most recent estimates of global emissions from deforestation (5.5 Pg CO_2_ yr^−1^ including freshwater peatlands) [1], or offset 12–80% of the carbon sink in the ocean's continental shelves globally (1.26 Pg CO_2_ yr^−1^) [58]. The lost annual sequestration potential of coastal ecosystems, which is considerable, would push these estimates higher [22].

Worth noting is that these estimates account only for changes in ecosystem C *in situ*, and do not account for possible exchanges among different ecosystems – e.g., the transfer of C from one system in another, which would effectively reduce the atmospheric emissions result. The degree to which some disturbed blue carbon is merely redistributed (e.g., exported from a disturbed mangrove to adjacent seagrass) just means that the true value of global emissions may be more toward the lower end of our uncertainty range, which assumes as much as 75% retention of near-surface carbon. While the amount of C transferred to other habitats is likely to be small compared to the C gas emissions described here, we recommend care be taken when aggregating carbon budgets across multiple habitats should they include assumptions on the transfer and deposition of carbon from one habitat to another.

Although tidal marshes, mangroves, and seagrasses occupy only a thin coastal fringe, they play a disproportionally large role in land-use carbon gas emissions. For example, compared to the highly publicized loss of tropical forests, the combined area of the three coastal ecosystems equates to only 2–6% of tropical forest area but contributes up to an additional 19% over current estimates of deforestation emissions. Disturbance of the carbon stored in the biomass and top meter of sediment in a typical hectare of mangrove could contribute as much emissions as three to five hectares of tropical forest [24], [28], [43], [48], [59]. Even a hectare of seagrass meadow, with its small living biomass, may hold as much near-surface carbon as a hectare of tropical forest [47], [48], [59].

The emissions estimates derived here are considerably higher than previous estimates of the potential greenhouse impact of coastal ecosystem loss that have only considered lost sequestration potential. Bridgham et al. [16] estimated that the destruction of mangroves and tidal marshes has resulted in reduced sequestration of 0.076 Pg CO_2_ per year. Pidgeon [60] estimated that 0.003 Pg CO_2_ per year of sequestration potential are lost due to current rates of mangrove and seagrass loss. Irving et al. [22] provided an analysis of the large sequestration potential of restoring degraded coastal ecosystems. Those studies focused on the annual new sequestration that is lost (gained) when the ecosystem is converted (restored). Our estimates focus on the loss of carbon stocks in coastal ecosystem sediments that have accumulated over hundreds to thousands of years and are lost, upon disturbance, within a period of decades [23]. These emissions (summed over all converted area and assuming a relatively constant or increasing conversion rate globally) are additional to the lost sequestration potential just referenced.

### Economic impacts

Combining the uncertainty range in emissions with a central estimate for the social cost of carbon gas emissions of $41 per Mg of CO_2_, we estimate the current global cost of coastal ecosystem conversion to be between $6.1 and $42 billion incurred annually (Table 1). The range would be even wider if we considered the full range of SCC values from $7–81 [57]. However, even at the low end of the range there is relatively high economic value in maintaining sediment carbon beneath coastal ecosystems and out of the atmosphere. The high ongoing cost of coastal ecosystem loss also supports the conclusion of Irving et al. [22], that management efforts focused on reducing coastal habitat loss may be more beneficial than the extensive restoration efforts being conducted in many regions which have smaller carbon benefits.

Around the globe, coastal ecosystems are lost because market forces give landowners incentive to profitably convert habitat. Elsewhere, ecosystems are lost because governments have been unwilling or unable to enforce clean water regulations and other measures that would help guarantee the continued ecological sustainability of these systems. There are, however, only a few mechanisms currently in place that would pay landowners, managers, or governments to protect the carbon stored in coastal ecosystems,

The cost of coastal ecosystem protection includes the expense of creating and managing protected areas, improving water quality, and particularly the opportunity costs of foregone alternative uses (e.g., aquaculture, real estate development). These costs can be quite high in some cases; therefore strong economic incentive would be required to counteract conversion. Absent payment mechanisms for the protection of coastal carbon, the degradation and loss of coastal ecosystems will likely continue. The global economic consequences will exceed the social cost of increased greenhouse gases as the loss of the array of ecosystem services they provide, such as fishery nurseries, biodiversity support, and coastal protection have tremendous economic value in their own right [5], [8].

A global market for greenhouse gas emission reductions could help remedy this situation. Such “carbon markets” have been operating throughout the world since the adoption of the United Nation's Framework Convention on Climate Change's Kyoto Protocol, but there has been a very limited role for terrestrial carbon reductions (e.g., forests), and no role for carbon in coastal ecosystems. Recent efforts may create a global market opportunity for reduced emissions from deforestation and degradation (REDD+). Guidance on modalities relating to deforestation emissions [61] highlight the need to include significant carbon pools in forest reference emission levels and/or forest reference levels, or to otherwise provide reasons for omitting these pools. These guidelines may be applied to mangrove forests and their belowground carbon [62], providing one step toward inclusion of a major source of coastal blue carbon in such programs.

Other opportunities have been outlined to include coastal carbon management such as ecosystem conservation, restoration, and sustainable use into the UNFCCC [4], [63]. Nationally Appropriate Mitigation Actions (NAMAs) could be an opening for developing countries to reduce carbon gas emissions while increasing national capacity-building and data collection activities. The newly adopted definition of wetland drainage and rewetting under the Kyoto Protocol provides an incentive to account for anthropogenic greenhouse gas emissions and removals by Annex-I Parties [64]. These represent further potential mechanisms for reducing emissions of coastal blue carbon to the atmosphere.

### Remaining Uncertainties

Scientific understanding differs among the various coastal ecosystems. Based on a sensitivity analysis within the Monte Carlo simulations, the largest contributions to uncertainty in emissions stemmed from wide published ranges for global area and conversion rates. Uncertainty is relatively high for emissions estimates for tidal marsh systems largely due to limited information on spatial extent, which had the widest influence on total emissions estimates of any input variable (accounting for 30% of total uncertainty). For mangroves, global area is better quantified, but uncertainty in conversion rates is substantial and had a large influence on total emissions estimates (18%). For seagrasses, the range in conversion rate was the most important influence on total uncertainty (14%). The proportion of C lost when converted had variable influence: the range for tidal marshes contributed only 2% total uncertainty, that for seagrasses contributed 9%, and that for mangroves contributed 18%. The value is largest for mangroves because they contain the largest near-surface C stocks. However, because of the limited number of studies of whole-ecosystem blue carbon stocks in these systems, we did not apply ranges in carbon stock estimates, focusing instead on the proportion released as applied to the best available central estimates. Further studies across a broad geographic range will allow development of likely ranges of C stocks and a more complete accounting of the uncertainty in blue carbon (gas) emissions. Overall, the most important information needs relevant to moving forward with blue carbon conservation (e.g., REDD+) include better quantification of the global area of tidal marshes and seagrasses, the actual areal conversion rates of mangrove and seagrass ecosystems, and the fate of blue carbon when disturbed in all systems.

We focused on potential CO_2_ emissions from conversion of standing stocks (in this case, determined by areal rates of conversion) and did not address the separate effects of changes in background flux rates which have been covered in other analyses [16], [22], [57]. Lost annual C sequestration would effectively increase the emissions consequences of conversion. In the most saline systems (salinity >18), this is true even if the disturbance were to decrease emissions of CH_4_ [55]. In addition, common disturbances such as conversion to shrimp ponds may increase CH_4_ due to euthrophication [27]. Thus, we are potentially underestimating greenhouse consequences of conversion. In oligohaline tidal marshes, however, natural methane efflux is often present in undisturbed conditions and may decrease when altered [56], which diminishes the emissions consequences of conversion [65]. Although methane is a strong greenhouse gas, changes in its contribution within the context of blue carbon may be less than ∼10–15% of our estimates of increased CO_2_ emissions due to conversion [55], [65]. Nevertheless, further refinement of methane dynamics in response to ecosystem conversion remains a research need.

### Conclusion

We currently know that coastal ecosystems contain substantial quantities of blue carbon. To our knowledge this analysis is the first to a) combine the best available estimates of global area, conversion rates, and ecosystem C *stocks* (not simply lost sequestration potential) to estimate blue carbon emissions on a global scale; b) use an uncertainty analysis to identify key data uncertainties relevant to moving forward with conservation of blue carbon; and c) estimate the global economic impacts of blue carbon emissions. Our analysis suggests that the greenhouse consequences of conversion of these ecosystems are larger than previously appreciated, by as much as an order of magnitude. These emissions add considerably to existing estimates of land-use carbon gas emissions such as tropical deforestation. Although these ecosystems occur as relatively thin coastal fringes, the economic impacts of $US 6–42 billion per year are borne globally.

This analysis establishes bookends and highlights the likely importance of blue carbon conversion. Information available to support these estimates, however, has high uncertainty. New research is needed to improve our estimates of how much carbon is trapped in these ecosystems, how much carbon is released into the atmosphere by their conversion, and where on the planet carbon loss is occurring most rapidly. Our analysis incorporated widely varying inputs and therefore shows that, regardless of how the science is ultimately refined, the unaccounted carbon gas emissions from coastal conversions are quite likely very high.

While more natural science research is underway, the development of policies and protocols that allow existing and emerging carbon markets to compensate stewards for conserving these ecosystems and reducing the amount of carbon gas emissions to the atmosphere could move forward. If markets and policies are in place, emerging science can translate into action for coastal blue carbon. Such policies could have a significant impact on greenhouse gas emissions, and a transformational impact on the ecosystems themselves.
